# Physical Therapies for Delayed Onset Muscle Soreness: A Protocol for an Umbrella and Mapping Systematic Review with Meta-Meta-Analysis

**DOI:** 10.3390/jcm13072006

**Published:** 2024-03-29

**Authors:** Szczepan Wiecha, Paweł Posadzki, Robert Prill, Maciej Płaszewski

**Affiliations:** 1 Department of Physical Education and Health in Biala Podlaska, Faculty in Biala Podlaska, Jozef Pilsudski University of Physical Education, 00-968 Warsaw, Poland; 2 Department of Clinical Rehabilitation, University School of Physical Education in Kraków, 31-571 Kraków, Poland; 3 Kleijnen Systematic Reviews Ltd., York YO19 6FD, UK; 4 Faculty of Health Sciences Brandenburg, Brandenburg Medical School Theodor Fontane, 14770 Brandenburg an der Havel, Germany; 5 Center of Orthopaedics and Traumatology, University Hospital Brandenburg a.d.H., Brandenburg Medical School Theodor Fontane, 14770 Brandenburg an der Havel, Germany

**Keywords:** physical exercise, DOMS, muscle soreness, physiotherapy, recovery, evidence-based, meta-research

## Abstract

Background: Delayed onset muscle soreness (DOMS), also known as exercise-induced muscle damage (EIMD), is typically caused by strenuous and/or unaccustomed physical exercise. DOMS/EIMD manifests itself in reduced muscle strength and performance levels, increased muscle soreness, swelling, and elevated levels of inflammatory biomarkers. Numerous randomised controlled trials (RCTs) and systematic reviews (SRs) of a wide variety of physiotherapy interventions for reducing the signs and symptoms of DOMS/EIMD have been published. However, these SRs often arrive at contradictory conclusions, impeding decision-making processes. Objective: We will systematically review the current evidence on clinical outcomes (efficacy, safety) of physiotherapy interventions for the treatment of DOMS/EIMD in healthy adults. We will also assess the quality of the evidence and identify, map, and summarise data from the available SRs. Method: Umbrella review with evidence map and meta-meta-analyses. MEDLINE, Embase, Cochrane Database of Systematic Reviews, Epistemonikos and PEDro will be searched from January 1998 until February 2024. SRs of RCTs of any treatment used by physiotherapists (e.g., low-level laser therapy, electrical stimulation, heat/cold therapy, ultrasound, magnets, massage, manual therapies) to treat DOMS/EIMD in healthy adults will be eligible. Narrative/non-systematic reviews, studies of adolescents/children and medically compromised individuals, of complementary therapies, dietary, nutritional, or pharmacological interventions, as well as self-administered interventions, or those published before 1998, will be excluded. AMSTAR 2 will be used to evaluate the methodological quality of the included SRs. Corrected covered area, will be computed for assessing overlaps among included SRs, and an evidence map will be prepared to describe the credibility of evidence for interventions analysed in the relevant SRs. Discussion: DOMS/EIMD is a complex condition, and there is no consensus regarding the standard of clinical/physiotherapeutic care. By critically evaluating the existing evidence, we aim to inform clinicians about the most promising therapies for DOMS/EIMD. This umbrella review has the potential to identify gaps in the existing evidence base that would inform future research. The protocol has been registered at PROSPERO (CRD42024485501].

## 1. Background

Strenuous and/or unaccustomed physical exercise of sufficient intensity and/or duration involving eccentric muscle contractions (where the muscle lengthens while under tension) typically induces temporary skeletal muscle micro-damage [1,2]. It causes a sequential release of tumour necrosis factor-alpha (TNF-alpha), interleukin (IL) 1 beta, IL-6, and IL-1 receptor antagonist (IL-1ra), as well as elevated levels of creatine kinase (CK) and lactate dehydrogenase (LDH) in the blood [3]. That localised inflammation clinically manifests itself in delayed onset muscle soreness (DOMS) and is characterised by increased pain and swelling and decreased muscle function [4]. The signs and symptoms peak between 24 and 72 h after the initial bout of exercise. Muscular micro-damage as well as biochemical and inflammatory phenomena are often referred to as exercise-induced muscle damage, EIMD, whereas subjective symptoms such as feelings of muscle tension and pain are typically labelled as DOMS [5]. Nonetheless, the literature is not consistent and the term DOMS is used both to label physiological mechanisms (like EIMD) [6] and their outcomes [7,8]. Therefore, we use both terms interchangeably.

The DOMS/EIMD phenomenon may lead to signs and symptoms which range from mild and negligible to severe pain and discomfort and impaired muscle performance capacities in elite and competitive sports [9], but may also be responsible for postural dysfunctions [10]. There are numerous approaches to both prevent and treat DOMS including non-steroidal, anti-inflammatory drugs (NSAIDs) [6,9], as well as dietary [11,12], complementary and alternative medicines (including herbs) [13,14], or physiotherapy (PT) [15]. With regards to the latter, those typically include (manual) massage, lymphatic drainage, heat or cold therapy (cryotherapy, ice/cooling, extreme cold), stretching, vibration, low-level laser therapy, low-intensity exercise, compression therapy, flossing, or taping, among others. Those therapies are purported to increase blood circulation and lymphatic flow to the muscle tissue, decrease oedema and muscular tone, facilitate the removal of lactic acid, and induce anaesthesia by releasing enkephalins and endorphins [16,17].

## 2. Rationale

A number of both randomised controlled trials (RCTs) and systematic reviews (SRs) evaluating the effectiveness of physical therapies for the treatment of DOMS have been published in recent decades [1,4,5,6,8,15,18,19,20,21,22,23]. These studies often arrive at contradictory conclusions, creating confusion and adding to the existing uncertainties which in turn makes clinicians’ decisions difficult [8,15,24]. 

A number of reports seem outdated, both as regards their findings and methods [6,25,26], whereas others should be analysed as regards their credibility [27,28,29]. To the best of our knowledge, none of those studies convincingly demonstrated the effectiveness of any PT intervention, and no tertiary study [30] has been published on this topic.

By synthesising evidence from SRs evaluating multiple treatments, umbrella reviews (URs) (or overviews of systematic reviews, or else overviews of reviews [31,32]) provide a useful source of information for researchers, athletic trainers, exercise physiologists, educators, policy-makers, and clinicians alike. By combining data from various meta-analyses addressing the same interventions or outcomes, meta-meta-analyses provide additional quantitative insights to URs, by pooling the data from the meta-analyses included in an UR [33,34]. Our UR will also involve a mapping exercise and a critical appraisal of the available evidence [35]. To the best of our knowledge, this would be the first UR to comprehensively evaluate the strength and validity of research evidence concerning various physiotherapeutic interventions for DOMS. Currently, no tertiary studies or standardized protocols are available in this domain. Consequently, the development of an evidence map could significantly aid therapists, coaches, clinicians, and athletes in selecting effective treatments for DOMS. An evidence map offers a thorough methodology for synthesizing and presenting relevant evidence to a specific research question or topic. It provides a visual representation or structured database summarizing the distribution of available evidence and plays a crucial role in assisting policymakers, researchers, and practitioners in grasping the current state of evidence related to a particular topic. By providing a clear overview, evidence maps facilitate evidence-based decision-making processes [35]. The objective for providing an evidence map in our UR is also to facilitate a comparison of different physiotherapies aimed at reducing DOMS, analysed in the eligible SRs, in a visually accessible format, aiding informed decision-making.

## 3. Methods

We will include SRs of RCTs of any type of intervention used by PTs in healthy adults (>18 and <65 years) at any level of physical activity/sports performance with DOMS/EIMD against any type of comparator, evaluating efficacy and safety outcomes. We will include SRs evaluating PT interventions applied post-exercise, in individuals experiencing DOMS and aimed at alleviating the signs and symptoms of it. SRs with quantitative pooling, i.e., meta-analyses, will be included for meta-meta-analysis, whereas SRs with qualitative syntheses will be included for descriptive analysis only. To be included in this UR, an SR must fulfil the minimum methodological criteria as defined by the Centre for Reviews and Dissemination guidance, such as reproducible search strategy, clearly stated eligibility criteria, and methodological quality/risk of bias appraisals of included studies [36]. 

SRs evaluating preventive PT interventions will not be included. We will also exclude SRs synthesising data from non-RCTs as well as narrative or non-systematic reviews [37,38] or those published before 1998 (to be used in the background/discussion section). This cut-off date was chosen based on the earliest indexed SR on DOMS/EIMD at the Physiotherapy Evidence Database, PEDro. SRs of medically compromised individuals, or those evaluating complementary therapies (e.g., acupuncture, reflexology, herbal medicine, homeopathy), dietary supplements/nutritional interventions (e.g., amino acids, creatinine, beetroot, caffeine, curcumin, l-carnitine, omega-3 fatty acids, pomegranate, spirulina, vitamins C, E), or pharmacological interventions (e.g., cyclo-oxygenase 2 inhibitors, non-selective nonsteroidal anti-inflammatory drugs) will also be excluded. Self-administered interventions (e.g., compression garments, foam rolling) will not be considered eligible [39,40]. SRs addressing children and adolescents will be excluded given distinct characteristics of inflammation and muscle remodelling processes in those populations [41]. See Table 1 for the detailed eligibility criteria.

### 3.1. Literature Searches

We will search the following databases: MEDLINE (Ovid), EMBASE (Ovid), Cochrane Database of Systematic Reviews, CDSR (Wiley), Epistemonikos, and PEDro. We will check the reference lists of the retrieved SRs for any additional studies. We will also run forward citation tracking, i.e., checking whether a work has been cited after its publication. A draft search strategy for MEDLINE is available in the Supplementary File S1. We will adapt the search strategy for each of the databases separately. Depending on databases, searches will combine, e.g., Medical Subject Headings terms, EMTREE, and terms appearing in the titles/abstracts. We will identify the relevant search terms through a discussion with the review team and by reviewing background literature. Searches will not be limited by language or publication status (unpublished or published). However, the searches will be limited by date, i.e., January 1998–January 2024. 

### 3.2. Handling of Citations

We will download identified references into EndNote 20 bibliographic management software for further handling (EndNote X9, Clarivate, PA, USA).

#### Quality Assurance within the Search Process

The main MEDLINE strategy will be independently peer-reviewed by the second author. The peer review process will be informed by the PRESS Peer Review of Electronic Search Strategies 2015 Guideline Statement [42].

### 3.3. Study Selection

Titles and abstracts thus identified will be independently screened by the two reviewers. During this initial phase of the screening process, we will exclude any references which do not meet the inclusion criteria. We will obtain full papers for all the remaining references. These will then be independently examined in detail by the same two reviewers to determine whether they meet the criteria for inclusion in the review. We will record details of those studies assessed during the full paper screening phase, including any reasons for exclusion. With respect to both screening stages, we will resolve any discrepancies through a discussion, or a third reviewer will act as an arbiter, if necessary. 

### 3.4. Data Extraction

Data extraction sheets will be individually designed and piloted in consultation with the research team, using Microsoft Excel^®^ (version 2021, Microsoft Corporation, Redmond, WA, USA). The types of data to be extracted will include details of the populations, interventions (dose, frequency, intensity, and duration), control groups, confounders and/or co-interventions, outcomes (types of), and effects estimates/overall results. We will also extract the date of the last database searches, number of RCTs, total sample size, risk of bias (RoB) in primary studies, whether meta-analysed (or not), the review authors’ conclusions (and direction of conclusion, i.e., positive, negative, equivocal), and whether any adverse effects (AEs) were reported. We will contact the authors of the included SRs for any missing data. Data extraction will be performed by the two independent reviewers. Any discrepancies will be resolved through a discussion with a third reviewer.

### 3.5. Quality Assessment

The methodological quality of each included SR will be assessed using the AMSTAR 2 tool to ensure that the conclusions and findings of the reviews are based on the best available evidence [43]. The AMSTAR 2 tool serves as a prevalent method for meticulously assessing reviews, comprising a total of 16 inquiries. Respondents evaluate each question as either ‘yes’, ‘no’, or ‘partial yes’. Among these, seven questions are deemed critical, while the remaining nine are non-critical in nature. The assessment outcome categorises the overall quality of a systematic review (SR) into four tiers: ‘high’, ‘moderate’, ‘low’, or ‘critically low’ [43]. Two reviewers will independently perform the assessments. Any discrepancies will be resolved through a discussion with a third reviewer.

### 3.6. Narrative Synthesis

A narrative summary of all the included SRs will be presented in a format to be agreed upon with the research team. This will include a summary of the characteristics and results of each SR. These findings will be discussed with reference to the certainty of the recommendations formulated, according to the individual AMSTAR 2 assessments. This will include the identification of any risks which may introduce bias into the data or any factors which may limit the credibility, reliability, and/or generalisability of the findings. The data will be summarised using text and, where relevant, accompanying tables and figures (graphs, bar charts, etc.).

### 3.7. Quantitative Synthesis

To estimate the effect size and the variance of each study, we will use the metaumbrella package (version 1.0.9) for R (R Foundation for Statistical Computing, version v4.2.1, Vienna, Austria, https://www.rstudio.com (access on 5 February 2024)) [44].

We will use the restricted likelihood maximum estimator to quantify the between-study variance in the random-effects meta-analysis. We will assess the significance of pooled standardized mean differences (SMDs) and 95% CIs with adjusted Hedges’ g to address the potential overestimation of the true population effect size in smaller studies [45]. SMD calculations prove essential for comparing outcomes across studies that utilize varied scales or measurement instruments, facilitating a uniform comparison across different datasets [45]. The effect size categorization will be as follows: 0–0.19  =  negligible effect, 0.20–0.49  =  small effect, 0.50–0.79  =  moderate effect, and  ≥0.80  =  large effect [46]. Heterogeneity will be assessed using the I^2^ statistic with values ≥75% indicating high, >50% moderate, and >25% low heterogeneity, respectively [47]. In instances where SRs will not report sufficient data by observation time and will present a combined effect, we will extract pertinent data, i.e., means, standard deviations, and 95% confidence intervals. If no figures are available, we will extract the data from the available graphs using WebPlotDigitizer software (Webplotdigitizer: Version 4.6, Pacifica, CA, USA). Funnel plot asymmetry (small study effects) will be evaluated with Egger’s test [48]. Finally, we will use the combined method (TESSPSST) of the proportion of statistical significance test (PSST) and test of excess statistical significance (TESS) to measure whether there is an excess of studies with statistically significant results [49]. Excess significance will be considered at *p* < 0.10. 

We will also calculate the amount of overlap of primary trials in the included SRs, i.e., corrected covered area (CCA) using the Pieper et al. formula, which will be calculated by the following formula [50,51]: CCA = (N − r)/(r•c − r) where, “N” represents the total number of included publications, considering the double counting of overlapped trials, “r” denotes the number of trials included, while “c” signifies the number of meta-analyses conducted. 

The interpretation of the CCA values will provide insights into the extent of overlap in the following manner: 0–5 will indicate slight overlap, 6–10 will suggest moderate overlap, 11–15 will signify high overlap, and values exceeding 15 will suggest very high overlap. Overlap analyses will be conducted for each therapy independently within the identified SRs, regardless of whether a meta-analysis was conducted. After a thorough examination of each paper included in the individual SRs, only duplicated RCTs within the PICO criteria defined in our umbrella review will be considered in the calculation. We will use the Graphical Representation of Overlap for OVErviews.

(GROOVE tool) for a graphical representation of overlap [52].

### 3.8. Subgroup Analyses

We will conduct the following subgroup analyses in this review where possible:

− By types of interventions, e.g., cold water immersions or type of manual/massage therapy;
− By the interventions’ intensity or duration or frequency, e.g., ice massage vs. whole body cryotherapy;
− By time of intervention, e.g., before versus after exercise;
− By country, i.e., physical therapists (mainly United States) vs. physiotherapists (e.g., EU, Australia);
− By athletic discipline, e.g., long distance (marathon) runners vs. short distance runners;
− By gender, i.e., males versus females;
− By age groups, e.g., young adults (18–44 years) versus midle-aged adults (45–64 years);
− By physical activity level, e.g., sedentary vs. active.
− By types of control groups, e.g., passive versus active.
− By the review’s risk of bias, i.e., low versus high risk of bias;
− By the primary studies’ risk of bias, i.e., low versus high risk of bias.

We acknowledge that there are further subgroup analyses that can potentially be performed. 

### 3.9. Evidence Map

An evidence map will be created using the following four dimensions [35]:

− The size of each bubble corresponds directly to the number of cases in the experimental groups among studies included in the respective SRs after excluding overlapping RCTs;
− Bubbles are colour-coded, with red indicating a very low percentage (0–15%) and blue indicating a high percentage (40%) of studies at an overall low risk of bias assessed in the respective SRs (e.g., PEDro scale, Cochrane ROB/ROB-2 tools);
− Therapies will be categorized according to the effect size (standardized mean difference (SMD)/adjusted Hedges’ g); only when the effect size favours the intervention groups. When the effect favours the controls or is not statistically significant (*p* > 0.05) it will be classified as NS on the Y-axis;
− Therapies will be grouped into five personalized categories as described by Fusar-Poli and Radua (2018) [33]. A therapy will be considered Convincing (Class I) if the case count exceeds 200, the *p*-value is less than 0.000001, I^2^ is less than 25%, Egger’s test yields a *p*-value greater than 0.1, the test for excess of significance bias (ESB) shows a *p*-value greater than 0.1, and the meta-analysis is powered at least 80% to detect an SMD of 0.2. A classification of Highly Suggestive (Class II) will apply when the case count is over 100, the *p*-value is less than 0.0001, I^2^ is between 25% and 50%, Egger’s test result is greater than 0.05, the ESB test result is greater than 0.05, and the meta-analysis has a power less than 80% to detect an SMD of 0.2 but greater than or equal to 80% to detect an SMD of 0.4, provided that Class I criteria are not met.

### 3.10. Study Reporting Guideline

We will report the study according to the criteria of Preferred Reporting Items for Overviews of Reviews (PRIOR) [53]. The PRIOR statement provides guidelines for synthesizing systematic review evidence to address various types of questions related to efficacy and effectiveness. These guidelines emphasize transparency and completeness in reporting, aiming to enhance the quality and reliability of overviews of reviews (umbrella reviews/systematic overviews of reviews).

#### Protocol Registration and Reporting

The protocol has been registered at PROSPERO (CRD42024485501] and prepared in accordance with the Preferred Reporting Items for Systematic Review and Meta-Analysis Protocols (PRISMA-P) reporting guideline [54].

## 4. Discussion

This will be the first umbrella review coupled with a meta-meta-analysis where we will summarize the efficacy of specific therapies used by physiotherapists for the relief of post-exercise muscle pain in the course of the so-called delayed muscle soreness syndrome (DOMS). Reanalyzing primary data from individual systematic reviews will enable the direct comparison and critical assessment of therapies, using established classifications concerning the strength of scientific evidence for better clarity. The data, derived from analyzing participant numbers, heterogeneity, *p*-values, publication bias, and excess significance bias, will be graphically displayed, showcasing the classifications achieved. Furthermore, we will compare the effect size with that of control groups using Hedges’ g SMD.

The various therapies are predominantly administered post-exercise, with follow-up ranging from immediately to several days afterward. Prolonged observation post-exercise may introduce increased error due to accumulating confounding factors. Therefore, observations are limited to 96 h post-exercise, with most studies noting changes between 24–72 h, correlating with the typical pattern of pain fluctuation in DOMS [6].

Some SRs also evaluate soreness within the immediate post-exercise time (up to 6 h), a period unlikely to represent DOMS. Pain gradually increases post-exercise, associated with inflammatory processes. Conversely, pain during or immediately after exercise is often linked to exercise-induced metabolic imbalances [6]. 

In our initial search and analysis of SRs to date, we have identified several potential groups of therapies that may have an impact on DOMS. The following actions and effects are attributed to the identified interventions:

Cold therapy, including local cooling methods like gels, compresses, towels, and automatic cuffs, as well as activities such as submerging in water below 15 °C, ice massage, cold whirlpool, and air-pulsed cooling [19,24]. It is widely recognized for its immediate impact on reducing inflammation and pain through vasoconstriction and decreased metabolic activity [6]. Notably, cold therapy emerges as one of the most prevalent therapeutic strategies employed [14,15,20].

Massage therapy has been advocated for its ability to enhance blood flow, reduce muscle tension, and promote relaxation [55]. While its immediate effects on performance may be limited, its role in improving flexibility and reducing soreness post-exercise is supported by anecdotal and some scientific evidence [56]. However, the effectiveness can vary and in some contexts it may temporarily reduce muscle function [56].

Vibration therapy introduces an interesting dimension by potentially accelerating recovery through increased muscle activation and blood circulation [57]. This modality might stimulate muscle fibers and enhance neuromuscular performance, which could be particularly beneficial during the recovery phase [58].

Active recovery involving low-intensity exercise post-exertion promotes blood circulation and toxin removal, aiding in the recovery process. This method leverages the body’s natural physiological processes to facilitate recovery and reduce soreness [59].

Stretching, often integrated into cooldown routines, aims to improve flexibility and reduce muscle stiffness. While its effectiveness in preventing DOMS is debated, it remains a staple in many athletic and rehabilitation programs [18].

Kinesio taping, which has gained popularity for its supposed ability to support muscle function, reduce pain, and facilitate lymphatic drainage, could assist in eliminating muscle metabolites and decreasing inflammation, hinting at its potential efficacy in facilitating recovery from DOMS [60]. 

Electrical stimulation offers a broad spectrum of applications, from pain management to muscle stimulation. Its use in DOMS treatment focuses on enhancing blood flow, reducing pain, and supporting muscle function, with various modalities tailored to specific needs [61].

Phototherapy, including low level laser and LED therapy, targets cellular mechanisms to reduce inflammation, promote healing, and decrease pain. By influencing mitochondrial activity and reducing oxidative stress, it presents a promising avenue for enhancing muscle recovery and reducing soreness [62].

These therapies provide diverse approaches to manage and alleviate DOMS symptoms, providing athletes with multiple options for recovery and injury prevention. Ongoing research is crucial to fully understand the mechanisms, optimize application, and integrate these therapies effectively into sports medicine and rehabilitation strategies.

A particular challenge is the growing amount of published systematic reviews and other secondary evidence, their methodological rigour, and credibility of their findings [28,63]. Currently, there is substantial methodological and clinical heterogeneity in SRs evaluating various PT modalities for DOMS. This diversity in terms of populations, interventions, comparators, outcome measures and study designs poses formidable challenges to clinicians, athletic trainers, exercise physiologists, policy-makers, researchers, and educators. By collating, synthesizing, and critically evaluating findings from a sizable number of SRs on DOMS/EIMD, we will produce a high level of robust and reliable evidence, reduce the existing uncertainties, and advance knowledge on the effectiveness of treatments utilised by PTs. Given the rapidly evolving field and the range of stakeholders involved, our UR is justified and necessary. From the methodological point of view, we will appraise the most common sources of bias and the certainty of the evidence, i.e., the degree of our confidence in the effect estimates derived from those SRs. We will explore a variation in effect sizes. With regard to safety, we will evaluate the reporting of any AEs including their number, severity, description, and causality. The potential role of any confounding factors such as diet (including hydration), lifestyle/behaviours, and sleep will also be analysed to further increase the generalisability of our findings. We will explore sources of heterogeneity, and suggest potential ways of reducing it. In a similar vein, we will investigate whether any of the methods or techniques have been appropriately standardised in terms of dose, frequency, intensity, and duration. Finally, we aim to formulate recommendations for research and clinical practice in DOMS. When conducting and reporting the results of this UR, we will strictly adhere to the Cochrane Handbook [64] and the PRIOR reporting guidelines to minimise any potential sources of bias.

## 5. Limitations

We recognise potential limitations associated with the numerical data due to differences in the methodological quality of the SRs which we will include in our analyses. Therefore, we propose conducting an initial assessment of the reported results to address any discrepancies in the data extracted. The currently intended classification into subgroups may not cover all possibilities arising from the variability of the included SRs, e.g., with regard to the age of the subjects or the differences in the application details of the interventions. Therefore, our findings, based on the results of the meta-meta-analyses, may be limited to selected populations and may limited generalisability. Nevertheless, we anticipate the possibility of conducting additional analyses (meta-regression) to determine the potential impact of various factors, e.g., age, gender, training, etc., on the obtained differences.

## Figures and Tables

**Table 1:** Eligibility criteria.

Item	Included	Excluded
Population	Healthy adults of any level of physical activity and sports performance, with DOMS/EIMD	Children and adolescents, medically compromised and elderly individuals
Intervention	Physical therapies * e.g., low-level laser therapy, electrical stimulation, heat/cold therapy, ultrasound, magnets, massage or manual therapies, stretching, vibration, low-intensity exercise, compression therapy, flossing, taping	Complementary therapies, diet/nutrition, pharmacological interventions, self-administered interventions/self-employed techniques, preventive interventions ***
Comparator	Any	N/A
Outcome	pain severity or intensity, muscle soreness ^	N/A
Study design	Systematic reviews ^^ of randomised controlled trials	Narrative/non-systematic reviews
Timeframe	Published since 1998 **	Published before 1998

* performed/delivered by physiotherapists (after exercise); the list is not exhaustive. ** articles published before that date will not be included in the analysis but may be used narratively if they meet the remaining inclusion criteria; *** i.e., interventions administered before exercise; ^ defined as research articles with a reproducible search strategy, eligibility criteria and methodological/risk of bias assessments; ^^ measured with any measurement instruments, e.g., visual analogue scale, numeric rating scale; N/A—not applicable.

## Data Availability

Not applicable.

## Supplementary Materials

The following supporting information can be downloaded at: https://www.mdpi.com/article/10.3390/jcm13072006/s1, Table S1: Databases search strategy; File S1: PRISMA-P 2015 Checklist.
