# Supplementary material for: Physical Therapies for Delayed Onset Muscle Soreness: A Protocol for an Umbrella and Mapping Systematic Review with Meta-Meta-Analysis
Source: J Clin Med. 2024 Mar 29;13(7):2006. doi: 10.3390/jcm13072006 (PMC11012564; doi:10.3390/jcm13072006)
Supplement: Supplementary file 1 [file jcm-13-02006-s001.zip › Suplementary Table S1.pdf]

Table S1. Databases search strategy

**MEDLINE (Ovid)**
1. Exercise/ or Exercise Test/ or Physical Exertion/ or Athletic Injuries/<br>2. Muscle Rigidity/ or "Sprains and Strains"/ or Muscle Weakness/<br>3. (exercise and (muscle* adj2 (damage* or injur*))).tw.<br>4. (sore* adj3 musc*).tw.<br>5. (DOMS or EIMD).tw.<br>6. Delayed onset muscle soreness or (sore$ adj3 musc$ or pain$ adj3 musc$).tw<br>7. exercise induced muscle damage.tw<br>8. or/1-7<br>9. exp physiotherapy<br>10. exp Physical Therapy Modalities<br>11. physical therapy specialty/<br>12. physical and rehabilitation medicine/<br>13. or/9-12<br>14. systematic review or meta-analy$ OR metaanaly$ OR ‘meta analys$ OR meta-analys$.pt<br>15. 8 and 13 and 14
